# Remnant cholesterol and mild cognitive impairment: A cross-sectional study

**DOI:** 10.3389/fnagi.2023.1069076

**Published:** 2023-03-15

**Authors:** Qiaoyang Zhang, Shan Huang, Yin Cao, Guanzhong Dong, Yun Chen, Xuanyan Zhu, Wenwei Yun, Min Zhang

**Affiliations:** ^1^Department of Psychology, The Affiliated Changzhou No. 2 People’s Hospital of Nanjing Medical University, Changzhou, China; ^2^Department of Neurology, General Hospital of Northern Theatre Command, Shenyang, China; ^3^Department of Neurology, The Affiliated Changzhou No. 2 People’s Hospital of Nanjing Medical University, Changzhou, China

**Keywords:** remnant cholesterol, mild cognitive impairment, MCI, cognitive function, cholesterol

## Abstract

**Objective:**

Emerging evidence suggests that elevated remnant cholesterol (RC) correlates with several health conditions. To explore the association of plasma RC with MCI incidence and the relationship between plasma RC and different domains of cognition in MCI patients.

**Methods:**

Thirty-six MCI patients and 38 cognitively healthy controls (HC) were enrolled in the present cross-sectional study. Using total cholesterol (TC) minus high-density lipoprotein cholesterol (HDL-C) minus low-density lipoprotein cholesterol (LDL-C) as the formula for calculating fasting RC. Cognition was assessed using the Chinese version of the Montreal cognitive assessment (MoCA), Auditory Verbal Learning Test (AVLT), Digit Symbol Substitution Test (DSST), Trail Making Test (TMT), and Rey-Osterrieth Complex Figure Test (ROCF).

**Results:**

Compared to healthy controls, MCI patients had a higher level of RC, the median difference in RC levels between these two groups was 8.13 mg/dl (95.0%CI: 0.97–16.1). Concurrently, plasma RC level was positively associated with MCI risk (OR = 1.05, 95%CI: 1.01–1.10). Notably, elevated RC level was correlated with impaired cognition in MCI patients, such as DSST (*pr* = −0.45, *p* = 0.008), ROCF- Long Delayed Recall (*pr* = −0.45, *p* = 0.008), AVLT-Immediate Recall (pr = −0.38, *p* = 0.028), and TMT-A (*pr* = 0.44, *p* = 0.009). Conversely, no significant correlation was found between RC and the AVLT-Long Delayed Recall test.

**Conclusion:**

This study found that plasma remnant cholesterol was associated with MCI. Further large longitudinal studies are needed in the future to confirm the results and clarify the cause-and-effect relationship.

## Introduction

Dementia is defined as a significant decline in cognition that interferes with independence and daily functioning. China accounts for approximately 25% of the world’s population with dementia (Jia et al., 2020). Mild cognitive impairment (MCI) is a transitional state between normal aging and dementia disorders, particularly Alzheimer’s disease (AD). Each year, approximately 10–15% of individuals with MCI develop dementia (Giau et al., 2019).

As elevated cholesterol in plasma has been linked to several health conditions, it may involve in the pathogenesis of MCI. For example, A large Chinese population-based study (*n* = 46,011) suggested hyperlipidemia as a risk factor for MCI (Jia et al., 2020). Another meta-analysis reported that elevated cholesterol levels in mid-life may increase the risk of cognitive impairment in late life, whereas higher levels of cholesterol in late life were not associated with dementia or cognitive impairment (Anstey et al., 2017). Thus, investigators believe that the relationship between cholesterol and MCI is age-dependent and mid-life hyperlipidemia is a risk factor for developing dementia or cognitive impairment at a later age.

In recent years, accumulating evidence suggests that remnant cholesterol (RC) in triglyceride-rich lipoproteins promotes residual atherosclerotic cardiovascular disease (ASCVD) risk after lowering low-density lipid cholesterol (LDL-C) to the recommended target (Saeed et al., 2018; Burnett et al., 2020; Langsted et al., 2020; Bruemmer and Cho, 2021; Chevli et al., 2022; Hao et al., 2022; Zheng et al., 2022). Remarkably, a large prevention study (*n* = 17,532) reported that RC predicts cardiovascular disease beyond LDL-C and apolipoprotein B in patients without known ASCVD (Quispe et al., 2021). RC is defined as total cholesterol (TC) minus LDL-C minus high-density lipid cholesterol (HDL-C). In the fasting state, RC includes very low-density lipoproteins (VLDL) and intermediate-density lipoproteins (IDL), and RC in the non-fasting state is composed of these two lipoproteins plus chylomicron remnants. Furthermore, except for cardiovascular disease, several studies reported that RC could predict stroke, hypertension, nonalcoholic fatty liver disease, diabetes mellitus, and aortic valve stenosis (Kaltoft et al., 2020; Jansson Sigfrids et al., 2021; Qian et al., 2021; Chen et al., 2022; Hu et al., 2022; Huang et al., 2022; Li et al., 2022).

Considering the wide connection between the diseases above and MCI, we hypothesized that plasma RC levels were also related to MCI. Therefore, in this study, we examined the association between plasma RC and MCI incidence and the relationship between plasma RC and different domains of cognitive performance in MCI patients.

## Materials and methods

### Ethics statement

This study was approved by the Institutional Review Board (IRB) of The Affiliated Changzhou Second People’s Hospital of Nanjing Medical University. All participants completed an informed consent form.

### Participants

A total of 38 MCI patients and 40 cognitively healthy controls (HC) participated in this cross-sectional study. However, 2 MCI patients and 2 HC subjects were excluded because of their negative RC levels calculated by formula. As a result, 36 MCI patients and 38 HC subjects were enrolled in our final analyses.

The MCI patients were recruited in the memory clinic of Affiliated Changzhou Second People’s Hospital of Nanjing Medical University in February–December 2021. The inclusion criteria included: (1) aged 50–70 years; (2) meeting the MCI criteria, based on the 2011 guidelines of the National Institute of Aging-Alzheimer’s Association workgroups (NIA/AA) (Albert et al., 2011). The exclusion criteria included: (1) having a substance use disorder except for nicotine; (2) personal or family history of severe psychiatric disorders; (3) a history of serious chronic medical conditions that may affect cognitive function, including liver and renal failure, hypothyroidism, cerebral infarction, cerebral hemorrhage; (4) a history of coronary heart disease or taking the prescribed lipid-lowering drug.

Community-dwelling volunteers aged 50–70 years who had a Montreal Cognitive Assessment (MoCA) score of 26 or higher were recruited as HC subjects. The exclusion criteria for HC subjects were the same as for MCI patients.

### Data collection

All participants provided sociodemographic data, health-related information, cognitive assessments, and blood sample for cholesterol analysis. All the data were collected in one day. Current smoking status was defined as at least 10 cigarettes/d for more than 3 years. Hypertension was defined as having a self-reported history of hypertension, SBP ≥ 140 mmHg, or DBP ≥ 90 mmHg, or taking any anti-hypertensive drugs. Diabetes was defined as any self-reported history, receiving hypoglycemic medication, fasting blood glucose ≥7.0 mmol/L, and/or OGTT ≥11.1 mmol/L.

### Cognitive assessment

Global cognition was assessed using the Chinese version of the Montreal cognitive assessment (MoCA). Meanwhile, different domains of cognition were measured as follows: (1) Memory: the Auditory Verbal Learning Test (AVLT), which includes immediate recall (AVLT-IR) and 20-min long-delayed recall (AVLT-LR); (2) Sustained Attention: the Digit Symbol Substitution Test (DSST); (3) Executive Function: the Trail Making Test (TMT), which includes part A (TMT-A) and part B (TMT-B); (4) Visuospatial Skill: the Rey-Osterrieth Complex Figure Test (ROCF), including immediate recall (ROCF-IR) and long-delayed recall (ROCF-LR). All scales were conducted by experienced investigators following the guidelines.

### Lipid measurement

Overnight fasting blood samples were drawn at 8 am during the medical check. Plasma lipids including TC, triglycerides (TG), HDL-C, and LDL-C were immediately enzymatically measured at the clinical laboratory on Roche Cobas 8,000 automatic biochemical analyzer with commercial reagents (Roche Diagnostics, Shanghai). RC was calculated by subtracting high-density lipoprotein cholesterol (HDL-C) and low-density lipoprotein cholesterol (LDL-C) from total cholesterol (TC):


RC=TC−[HDL−C]−[LDL−C]

We categorized the lipid measurements according to the Third Report of the National Cholesterol Education Program Expert Panel on Detection, Evaluation, and Treatment of High Cholesterol in Adults (ATP-III) (NCEP, 2002).

### Statistical analysis

Variables with a normal distribution were expressed as mean ± SD, while variables with a skewed distribution were expressed as median (interquartile range [IQR]). Categorical variables were expressed as frequencies (%). Demographic characteristics of the MCI and HC group were analyzed using independent samples t-test (normal distribution), Mann–Whitney *U*-test (non-normal distribution), and chi-square test (categorical variables).

Data Analyses with Bootstrap-coupled ESTimation (DABEST) were used to compare the differences in RC levels (Ho et al., 2019). By plotting the data as the median difference in RC levels between MCI patients and cognitively healthy controls, the Gardner-Altman estimation plots can help visualize the effect size.

Based on prior studies and theoretical considerations, we selected established risk factors for MCI (Liu et al., 2021; Wang M. et al., 2021). Thus, age, gender, education level, smoking status, hypertension, diabetes, and RC were entered into binary logistic regression analyses (Forward: LR). To examine whether impaired cognitive performance correlated with elevated RC in MCI patients, we tested their associations using partial correlation analyses controlling for age and education level. All analyses were performed with the statistical package R 4.2.0^1^ (R Foundation). The significance level was defined as *p* < 0.05 (two-sided).

## Results

### Demographic and clinical parameters of MCI patients and healthy controls

As shown in Table 1, there were significant differences between MCI patients and healthy controls in demographics, and cognitive performance, including age, education level, TMT-A, AVLT-IR, AVLT-LR, DSST, ROCF-LR, and MoCA (all *p* < 0.05). MCI patients had a worse performance at the cognitive subtests above.

**Table 1:** Comparison of characteristics between MCI patients and cognitively healthy controls.

Variable	Overall (*n* = 74)	HC (*n* = 38)	MCI (*n* = 36)	*P*
Sociodemographic and health-related characteristics				
Age	60.0 (56.0, 66.0)	58.5 (54.3, 63.0)	61.5 (57.0, 67.3)	**0.022** ^b^
Gender, *n* (%)				1^a^
Male	60 (81.1)	31 (81.6)	29 (80.6)	
Female	14 (18.9)	7 (18.4)	7 (19.4)	
Education, years	9.0 (8.0, 9.8)	9.0 (9.0, 12.0)	9.0 (7.0, 9.0)	**0.003** ^b^
Current smoker, *n* (%)				1^a^
No	40 (54.1)	21 (55.3)	19 (52.8)	
Yes	34 (45.9)	17 (44.7)	17 (47.2)	
History of hypertension, *n* (%)				0.889^a^
No	18 (24.3)	10 (26.3)	8 (22.2)	
Yes	56 (75.7)	28 (73.7)	28 (77.8)	
History of diabetes, (%)				0.876^a^
No	51 (68.9)	27 (71.1)	24 (66.7)	
Yes	23 (31.1)	11 (28.9)	12 (33.3)	
Lipid profiles				
Triglycerides, mg/dL	131.6 (100.1, 162.1)	127.6 (97.0, 162.1)	133.8 (111.9, 163.9)	0.36^b^
TC, mg/dL	157.4 ± 36.9	153.4 ± 36.1	161.7 ± 37.8	0.334^c^
LDL-C, mg/dL	85.8 ± 29.5	87.8 ± 28.8	83.7 ± 30.4	0.554^c^
HDL-C, mg/dL	39.9 ± 8.7	39.6 ± 9.2	40.2 ± 8.3	0.744^c^
RC, mg/dL	30.4 (20.1, 38.1)	26.3 (17.0, 33.4)	34.4 (26.0, 45.0)	**0.003** ^c^
Cognitive assessment				
TMT-A	50.7 ± 7.1	48.8 ± 6.7	52.7 ± 7.2	**0.02** ^c^
TMT-B	185.5 ± 23.8	180.4 ± 20.1	190.9 ± 26.3	0.058^c^
AVLT-IR	4.0 (3.0, 4.0)	4.0 (4.0, 5.0)	3.50 (3.0, 4.0)	**0.008** ^b^
AVLT-LR	5.0 (5.0, 6.0)	6.0 (5.0, 6.0)	5.0 (4.0, 5.0)	**<0.001** ^b^
DSST	26.7 ± 5.4	27.9 ± 5.9	25.3 ± 4.6	**0.042** ^c^
ROCF-IR	34.0 (33.0, 36.0)	35.0 (34.0, 36.0)	34.0 (32.8, 35.3)	0.182^b^
ROCF-LR	19.0 (16.3, 21.0)	19.0 (17.3, 22.0)	17.5 (15.0, 20.0)	**0.024** ^b^
MoCA	27.0 (25.0, 29.0)	29.0 (28.0, 29.0)	25.0 (25.0, 25.0)	**<0.001** ^b^

Bold indicated that the results were statistically significant. ^a^Comparison was tested by *χ*^2^-test. ^b^Comparison was tested by the Mann–Whitney *U* test. ^c^Comparison was tested by independent – sample *t*-test. HC, Cognitively healthy controls; MCI, Mild Cognitive Impairment; TC, Total Cholesterol; LDL-C, Low-Density Lipoprotein Cholesterol; HDL-C, High-Density Lipoprotein Cholesterol; RC, Remnant Cholesterol; TMT-A, Trail Making Test Part A; TMT-B, Trail Making Test Part B; AVLT-IR, Auditory Verbal Learning Test-Immediate Recall; AVLT-LR, Auditory Verbal Learning Test-Long Delayed Recall; DSST, Digit Symbol Substitution Test; ROCF-IR, Rey-Osterrieth Complex Figure Test – Immediate Recall; ROCF-LR, Rey-Osterrieth Complex Figure Test – Long Delayed Recall; MoCA, Montreal Cognitive Assessment.

### Lipid profiles of MCI patients and healthy controls

RC levels were significantly higher in MCI patients than in cognitively healthy controls (*p* < 0.05) (Table 1). However, these two groups had no significant difference in Triglycerides, TC, LDL-C, and HDL-C levels.

**Figure 1:**
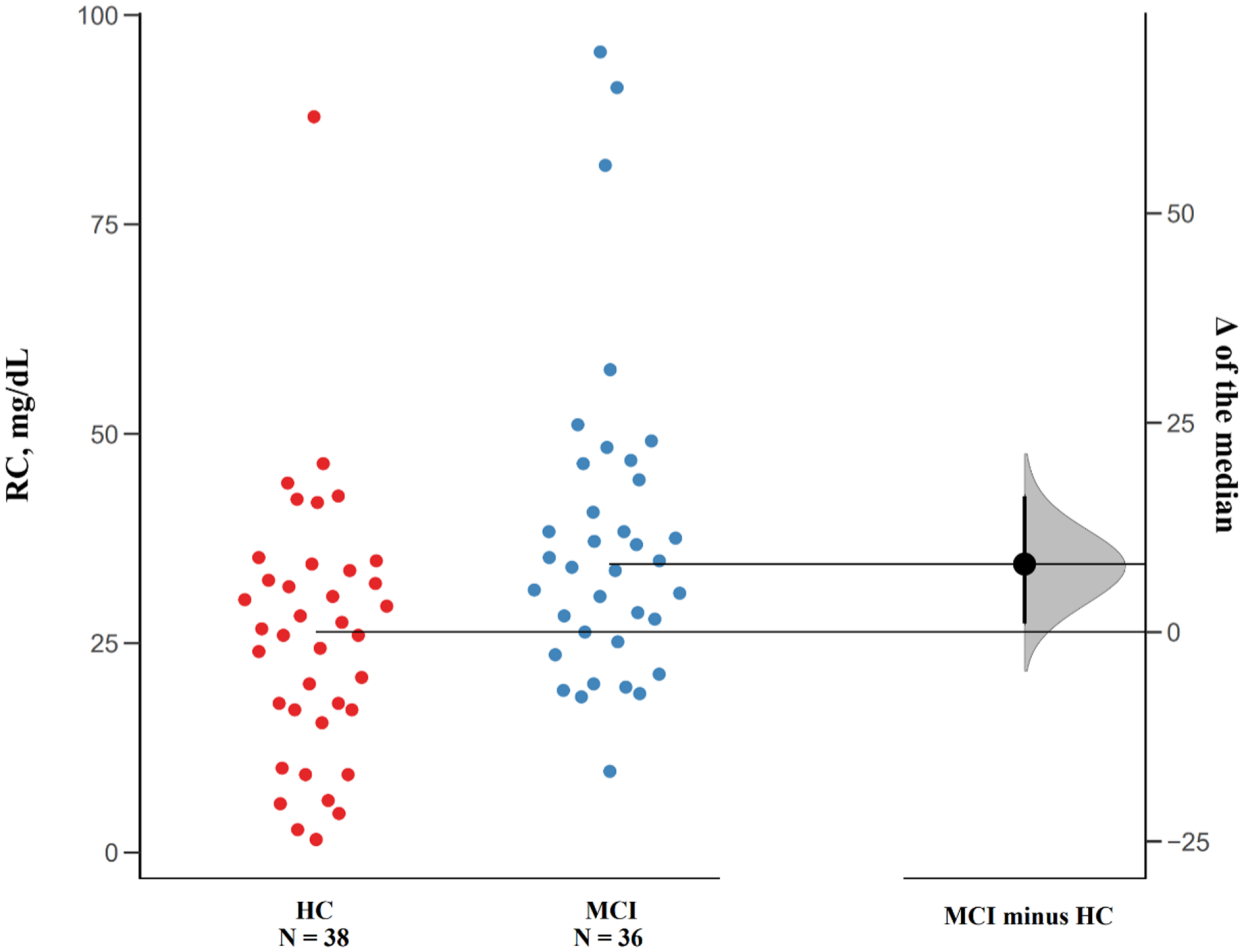
Median Differences of RC levels in MCI patients and cognitively healthy controls. Raw data points of both groups are plotted on the left panel, and the median differences are plotted on the right panel by using 5,000 bootstrapped resamples. The black dot in the right panel represents the median difference, the vertical error bar indicates 95% confidence interval, and the shaded area represents bootstrapped sampling error distribution.

In addition, the estimation plot of differences in RC levels was shown in Figure 1, the median difference in RC levels between MCI patients and cognitively healthy controls was 8.13 mg/dl (95.0%CI: 0.97–16.1).

### Affecting factors of MCI patients

As shown in Table 2, the related factors for MCI patients were as follows: age (OR = 1.18, 95%CI: 1.06–1.33), education level (OR 0.64, 95%CI: 0.45–0.84), and RC (OR = 1.05, 95%CI: 1.01–1.10). Specifically, RC was positively associated with the incidence of MCI, for every 1-unit (mg/dL) increase in RC, the incidence of MCI increased by 0.05 (95%CI: 1.01–1.10).

**Table 2:** Factors associated with MCI.

Variable	*B*	SE	Wald	OR	95% CI	*p*
Age	0.16	0.06	8.21	1.18	1.06–1.33	0.004
Education	−0.45	0.16	8.42	0.64	0.45–0.84	0.004
RC	0.05	0.02	6.15	1.05	1.01–1.10	0.013

RC, Remnant Cholesterol.

### Associations between RC and cognitive performance in MCI patients

**Figure 2:**
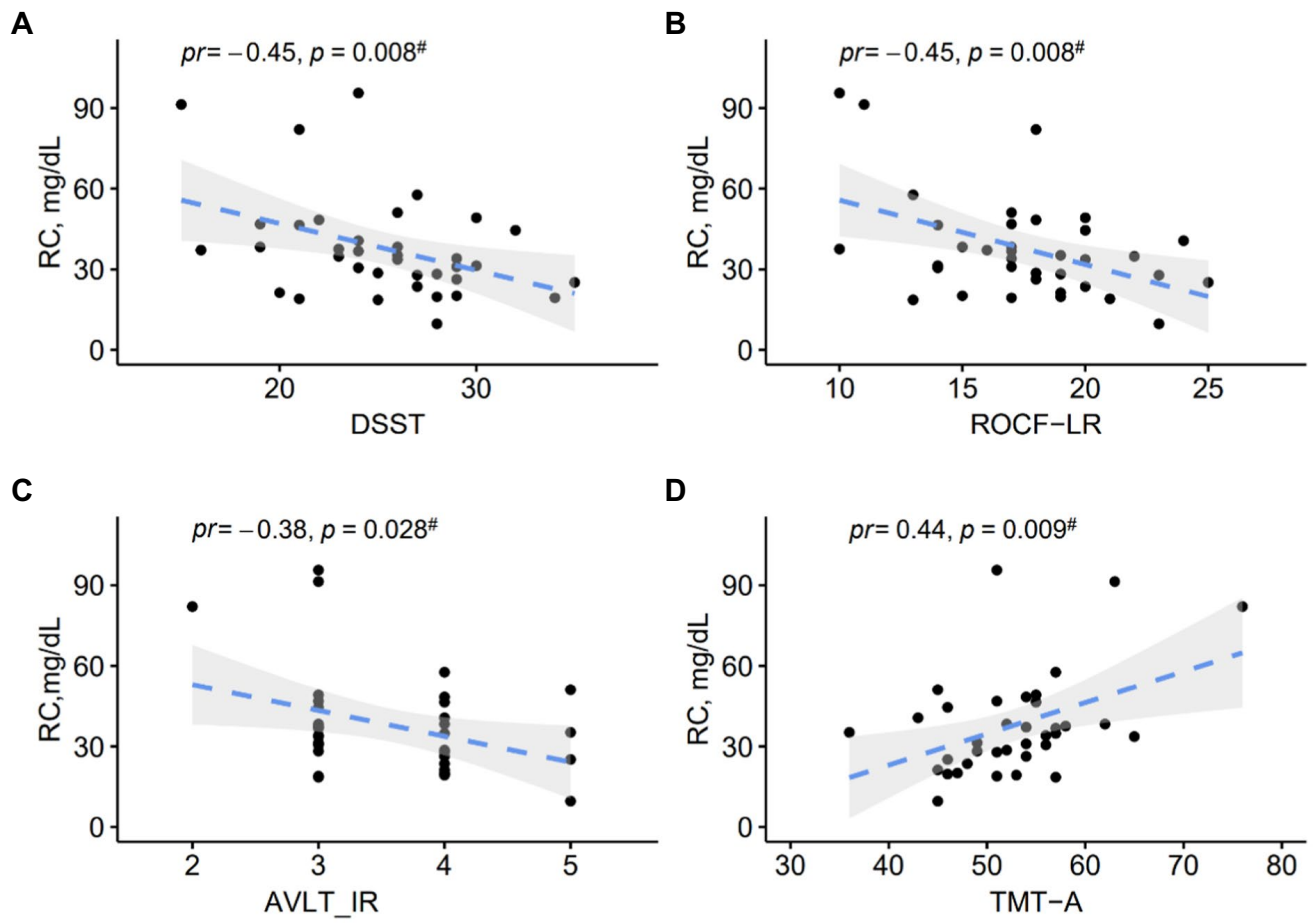
**(A–D)** Scatter plots show correlations between RC levels and cognitive performance in MCI patients. *pr*, partial correlation coefficient.

The partial correlation analyses in MCI patients were provided in Figure 2. Regarding the associations between RC levels and cognitive performance in MCI patients, we found significant negative correlations of RC levels with DSST (*pr* = −0.45, *p* = 0.008), ROCF-LR (*pr* = −0.45, p = 0.008), AVLT-IR (*pr* = −0.38, *p* = 0.028), and a positive correlation with TMT-A (*pr* = 0.44, *p* = 0.009). However, no significant correlation was found between RC and the AVLT-LR subtest.

## Discussion

In this study, we examined the relationship between plasma RC and MCI. The main results were (1) MCI patients had a higher level of RC than cognitively healthy controls; (2) Plasma RC level was positively associated with MCI risk; (3) RC level was related to impaired cognitive performance among MCI patients.

The mechanism of the association between RC and MCI remains unknown but biologically plausible. Vascular cognitive impairment (VCI), caused by cerebrovascular or cardiovascular diseases, is the second most common neuropathology of MCI (Aronow and Ahn, 2002; Smith, 2017; Gu et al., 2019). Specifically, cerebrovascular and cardiovascular diseases contribute to VCI *via* multiple types of vascular brain injury (e.g., infarcts, hemorrhages, white matter lesions, enlarged perivascular spaces, altered white matter microarchitecture, and disrupted network connectivity) (Smith, 2017). RC could also take part in the pathology of amyloid-positive MCI (Sagare et al., 2012). Experiments on cell cultures and animal studies suggested that the accumulation of cholesterol in neurons contributes to amyloid deposition in the brain by accelerating the cleavage of amyloid precursor proteins into amyloidogenic components, whereas cholesterol is kept low in neurons may inhibit Aβ accumulation (Toro et al., 2014; Wang H. et al., 2021). Furthermore, Reed B and colleagues (Reed et al., 2014) reported an association between persons’ serum cholesterol levels and cerebral β-amyloid (Aβ), with Aβ quantified using carbon C11-labeled Pittsburgh Compound B positron emission tomography.

We also found a wide range of relationships of RC with different domains of cognition, including executive function, visuospatial skill, immediate memory, and sustained attention, except for delayed memory. The small sample size might cause the lack of association between RC and delayed memory. Other possibilities could be the predominantly frontal impairment due to microvascular pathology, which needs to be confirmed in future studies. Besides, in the present study, we did not find differences in TG, TC, HDL-C, and LDL-C between MCI patients and healthy controls. Similarly, in a Chinese population-based study of older adults(*n* = 184), investigators reported that serum HDL-C was negatively related to the likelihood of MCI, without finding differences in serum TG, TC, and LDL-C between MCI patients and healthy controls (Wang M. et al., 2021). Conversely, in another Chinese case–control study (*n* = 227), plasma TC, TG, and HDL-C levels were reported to be associated with the risk of MCI, whereas LDL-C was not significantly different between the MCI group and controls (He et al., 2016). The inconsistent results may be influenced by different inclusion/exclusion criteria, and sample sizes.

Several limitations need to be acknowledged in the present study. First, the small sample size may impact the robustness of the study, thus further larger studies are needed to provide robust evidence for the relationship between plasma RC and MCI. Second, since the study design was cross-sectional, any causality of RC with MCI could not be explored. Third, residual confounders may exist due to several unmeasured factors such as marital status, BMI, physical activity, and depression. Lastly, the findings should be generalized with caution when considering our participants were only recruited from the memory clinic, population-based studies are needed in the future to verify the findings.

## Conclusion

In conclusion, to our best knowledge, this study is the first to identify a relationship between plasma remnant cholesterol and MCI. In addition, remnant cholesterol was associated with different domains of cognitive function in MCI patients. Larger longitudinal studies are needed in the future to confirm the results due to the small sample size.

## Data availability statement

The raw data supporting the conclusions of this article will be made available by the authors, without undue reservation.

## Ethics statement

The studies involving human participants were reviewed and approved by This study was approved by the Institutional Review Board (IRB) of The Affiliated Changzhou Second People’s Hospital of Nanjing Medical University. All participants completed an informed consent form. The patients/participants provided their written informed consent to participate in this study.

## Author contributions

QZ: conceptualization, methodology, software, investigation, formal analysis, and writing–original draft. XZ and YuC: data curation. GD: visualization and investigation. YiC and WY: resources and supervision. SH: visualization and writing–review and editing. MZ: conceptualization, funding acquisition, resources, supervision, and writing–review and editing. All authors contributed to the article and approved the submitted version.

## Funding

This study was supported by the General Program of Jiangsu Commission of Health (H2019051); the Elderly Program of Jiangsu Commission of Health (LKZ2022016).

## Conflict of interest

The authors declare that the research was conducted in the absence of any commercial or financial relationships that could be construed as a potential conflict of interest.

## Publisher’s note

All claims expressed in this article are solely those of the authors and do not necessarily represent those of their affiliated organizations, or those of the publisher, the editors and the reviewers. Any product that may be evaluated in this article, or claim that may be made by its manufacturer, is not guaranteed or endorsed by the publisher.

## Footnotes

^1^ http://www.R-project.org
